# Timeless Links Replication Termination to Mitotic Kinase Activation

**DOI:** 10.1371/journal.pone.0019596

**Published:** 2011-05-06

**Authors:** Jayaraju Dheekollu, Andreas Wiedmer, James Hayden, David Speicher, Anthony L. Gotter, Tim Yen, Paul M. Lieberman

**Affiliations:** 1 The Wistar Institute, Philadelphia, Pennsylvania, United States of America; 2 Merk Research Laboratories, West Point, Pennsylvania, United States of America; 3 Fox Chase Cancer Center, Philadelphia, Pennsylvania, United States of America; Duke University, United States of America

## Abstract

The mechanisms that coordinate the termination of DNA replication with progression through mitosis are not completely understood. The human Timeless protein (Tim) associates with S phase replication checkpoint proteins Claspin and Tipin, and plays an important role in maintaining replication fork stability at physical barriers, like centromeres, telomeres and ribosomal DNA repeats, as well as at termination sites. We show here that human Tim can be isolated in a complex with mitotic entry kinases CDK1, Auroras A and B, and Polo-like kinase (Plk1). Plk1 bound Tim directly and colocalized with Tim at a subset of mitotic structures in M phase. Tim depletion caused multiple mitotic defects, including the loss of sister-chromatid cohesion, loss of mitotic spindle architecture, and a failure to exit mitosis. Tim depletion caused a delay in mitotic kinase activity *in vivo* and *in vitro*, as well as a reduction in global histone H3 S10 phosphorylation during G2/M phase. Tim was also required for the recruitment of Plk1 to centromeric DNA and formation of catenated DNA structures at human centromere alpha satellite repeats. Taken together, these findings suggest that Tim coordinates mitotic kinase activation with termination of DNA replication.

## Introduction

Cell cycle progression is monitored by a series of checkpoint mechanisms that maintain genome integrity and cell viability [1]. Multiple checkpoint mechanisms have been defined for recognition and repair of DNA damage during interphase (e.g. G1/S and intra-S phase checkpoints) and chromosome dynamics during mitosis (e.g. spindle checkpoints) [2], [3], [4]. Relatively, little is know about mechanisms that coordinate the terminal stages of DNA replication with the entry into and progression through mitosis. Completion of normal DNA synthesis involves post-replication repair of small replication errors by translesional DNA polymerases and rescue of collapsed replication forks and double strand breaks by homologous recombination between sister chromatids. Many of these events are monitored by the ATM-Chk2 and ATR-Chk1 DNA damage checkpoint pathways, which regulate replication fork progression by directly modifying factors like PCNA and MCM subunits [2], [5], [6], [7]. Progression through G2 and mitosis requires the activation of a family of mitotic entry kinases, which include cyclin-dependent kinase 1 (CDK1), Polo-like kinase 1 (Plk1), and Aurora kinases [1], [8]. While these mitotic kinases can be inhibited by DNA damage checkpoint kinases, the mechanisms that promote their activation in response to normal termination of DNA replication are not well characterized.

Completion of S phase requires the processing of numerous replication structures including those formed by converging replication forks and replication fork barriers [9], [10], [11], [12]. Studies from yeast and other model organisms have identified a set of factors that regulate and monitor DNA replication during termination or fork pausing [9], [12], [13]. The Swi1–Swi3 complex from *S. pombe* has been implicated in recombination structure formation at termination sites and programmed pause sites for DNA polymerase [11], [14]. The programmed pause sites at the mating type switch locus and the ribosomal DNA repeats promote recombination. Subsequent studies have shown that Swi1–Swi3 travel with the replication fork during S phase and prevent the separation of the leading and lagging strand polymerases [15]. The *S. cerevisiae* orthologues of Swi1–Swi3, Tof1-Csm3, have been isolated in a stable replication pausing complex [16]. Genetic analysis of Tof1-Csm3, as well as Swi1–Swi3, have also been implicated in sister-chromatid cohesion [17]. It is not yet known how these replication fork protection factors promote sister-chromatid cohesion, an event associated with late G2 and early M phase, nor how replication pausing is coupled to homologous recombination (reviewed in [18]).

Timeless (Tim) and Tipin have been identified as the mammalian orthologues of Swi1 and Swi3, respectively [19]. Like their yeast counterparts, Tim and Tipin function in replication fork protection and genome stability [19], [20], [21], [22], [23]. Tim and Tipin form a stable complex that also includes Claspin, the mammalian orthologue of the mediator of replication checkpoint (Mrc1). Claspin is required for Chk1 and ATR activation during replication fork arrest [20], [24], [25]. In one study, depletion of Tim or Tipin resulted in reduced protein levels and cytoplasmic relocalization of Claspin [23]. Tim and Tipin associate with components of the replication fork, including Polδ, Polε, and multiple MCM subunits [21], [22], [26]. In mouse embryo fibroblasts, shRNA depletion of Tim produced a decrease in replication fork progression and elevated sister chromatid exchanges, presumably as a consequence of the increase in single strand DNA formation and chromatid breaks [27]. These studies establish that Tim-Tipin-Claspin function together as components of the mammalian and yeast replisome that are required for maintaining replication fork stability at programmed pause sites and during conditions of DNA damage.

The entry into mitosis following DNA replication is regulated through the interplay of CDK1-Cyclin B1, Plk1, and Aurora kinases [28]. CDK1 is activated by the dual specificity phosphatase CDC25 [29]. CDC25 activity can be amplified by a CDK1-dependent interaction with Plk1 [30]. Plk1 can be activated by Aurora A in G2, and this is controlled by the interaction of Aurora A kinase with one of several regulatory proteins, including TPX2, Ajuba, PAK1, Hef1, and hBora [8]. Plk1 can also interact with components of the replication fork, including MCM7 [31] and DDK [32]. The Xenopus paralogue, Plx, can bind and phosphorylate Claspin during adaptation response to DNA damage [33] and is required for chromosome DNA replication especially under conditions of stress associated with DNA polymerase inhibitor aphidicolin [34]. In a more recent study, Plk1 was implicated in a G2 DNA damage response checkpoint required for the stabilization of Claspin and dependent on the ubiquitin ligase APC/C^cdh^ and the phosphatase Cdc14B, a protein previously implicated in a later cell cycle control step during mitotic exit [35].

In addition to their functions in regulating mitotic entry and G2 DNA damage checkpoint, Plk and Aurora kinases are also components of the kinetochore that links the spindle microtubules to the chromosomal centromere during mitosis [28]. One of the major functions of Plk1 and Aurora B kinases at centromeres is the phosphorylation of histone H3 variant CENP-A on serine 7 [36], [37]. Aurora B kinase has also been implicated in the phosphorylation of histone H3 S10 at numerous other chromosomal sites during mitosis [38], [39]. Furthermore, Aurora B phosphoryaltion of H3 S10 has been implicated in regulating pericentric heterochromatin formation, which is essential for centromere function during DNA replication, sister-chromatid attachment, and kinetochore stability [37]. Centromeres contain programmed replication fork barriers at the alpha satellite repeats found in all mammalian centromeres [40]. Fork barriers, like those found in centromeres and ribosomal DNA repeats, are thought to promote formation of DNA recombination structures [12]. Yeast orthologues of Tim and Tipin maintain replication fork stability at fork barriers, but the precise mechanism of these proteins and the function of the fork barriers remain enigmatic. In this study, we show that Tim physically links mitotic entry kinases, Plk1 and Aurora A, to a replication fork barrier within the centromeric alpha satellite repeats in human cells. We also show that Tim is required for the timely activation of mitotic kinases and progression through mitosis.

## Materials and Methods

### Cell Culture and Cell Cycle Synchronization

HeLa, 293-T, and HCT116 cells were cultured in Dulbecco's modified Eagle's medium (DMEM) with 10% fetal bovine serum, glutamax, and antibiotics. For synchronization, cells were arrested at the G_1_/S boundary by incubation in the presence of 2.0 mM thymidine for 14–16 hrs twice with a 10 hr interval of growth without the drug. After the second thymidine arrest, cells were released into fresh DMEM media and harvested at the indicated time intervals.

### Antibodies

Rabbit anti-Tipin antibody and guinea pig anti-Tim antibody were raised against human peptides and described previously [19], [21]. All antibodies were affinity-purified. Antibodies from commercial sources were as follows: CDC2, PCNA, RPA34, Cyclin B1, CDC25C (Santa Cruz Biotechnology); α-tubulin and FLAG M2 (Sigma); γ H2AX, CENPA p7 (Cell Signaling Technology); Rabit-Tim (Bethyl Laboratories); Plk (Invitrogen); H3PhosphoSer10 (Millipore); AuroraA, AuroraB, Cdh1, SMC5, SMC6, MCM2, MCM3, MCM5, MCM7, BubR1 (Abcam).

### siRNAs and Transfection

Transfection of small interfering RNA (siRNA) duplexes was conducted by using Oligofectamine (Dharmacon, Inc), following manufacturers specifications. All siRNA oligonucleotides were purchased from Dharmacon. siRNA for Tim is 5′-GUAGCUUAGUCCUUUCAAAdTdT-3′. The sequences of control siRNA and on-target smart pool siRNA for timeless were reported in Dharmacon.

### Metaphase spreads

Cells were transfected with siRNAs and after 48 hr post-transfection, cells were treated with colcemid for 2.5 hrs. Cells were collected and metaphase spreads were prepared as described [41].

### Flag purification

Stable cells were made by co-transfecting 293T cells with pCMV-Flag-Timeless and puromycin resistant plasmids. Flag purification and peptide elution has been described previously [42].

### Immunoprecipitation

Cells were extracted with lysis buffer (20 mM Tris-HCL, pH 7.4, 1 mM EDTA, 0.1 mM EGTA, 2 mM MgCl_2_, 150 mM NaCl, 1 mM Na_3_VO_4_, 1 mM NaF, 20 mM sodium glycerophosphate, 5% glycerol, 0.5% TritonX100, 0.5% Nonidet P-40, 1× protease inhibitors (Simga), 1× Phosphatase inhibitors (Sigma), I mM PMSF and 2 mM NEM). After rotation for 30 min at 4°C, the lysate was centrifuged for 20 min at 15,000× *g*, and the supernatant was recovered. The cleared extracts were used for immunoprecipitation with antibodies as indicated.

### 2d gel electrophoresis

Cells were synchronized and collected as described above. DNA isolation and 2d gel electrophoresis were performed as described previously [43]. The membranes were hybridized with ^32^P labeled D17Z1 α satellite specific probe [40].

### Live cell imaging

GFP-H2B expressing Hela cells were transfected with siControl or siTimeless siRNA and cells were synchronized by double thymidine block and release. At 6 hr post-release, time-lapsed microscopy was started with 5 min intervals.

### Kinase assay

HCT116 cells were transfected with siControl or siTimeless siRNA and synchronized by double thymidine block. Cells were collected 6 hr after release from double thymidine block and lysed in lysis buffer (20 mM Tris-HCl pH 7.5, 150 mM NaCl, 1 mM EDTA pH 8.0, 0.1 mM EGTA, 0.1% TritonX100, 0.5% NP-40, 1 mM PMSF, 1 mM NaF, 20 mM sodium pyrophosphate, 20 mM sodium glycerophosphate, 1 mM sodium vanadate, 1× proteinase inhibitor cocktail (Sigma) and, 1× phosphatase inhibitor cocktail (Sigma)) on ice for 30 min with occasionally agitation. Cell debris was removed by centrifuge at 12,000 g for 5 min at 4°C. Total 50 ug of protein lysate was used for immunoprecipitation by incubating with 2 µg of rabbit anti-Plk, anti-AuroraA, anti-AuroraB, and anti- IgG at 4°C for o/n followed by protein A agarose conjugation at 4°C for 2 hours. The beads were washed two times with lysis buffer and once with kinase buffer (50 mM Tris-HCl pH 7.5, 5 mM MgCl2, 1 mM DTT and 0.01% NP40). The kinase reaction was performed at 30°C for 30 min in kinase buffer with or without 100 ng histone H3 (Roche) as substrate and 10 µCi of ^32^PγATP, 100 µM ATP. The reaction was stopped by adding 2× Laemmli buffer and boiled for 5 min before loading on a 4–20% SDS-PAGE. The gel was then exposed to X-ray film.

### FRET Assay

FRET assay to monitor Plk activity *in vivo* was performed essentially as described previously [44].

## Results

### Mitotic kinases physically associate with Tim protein complex

**Figure 1:**
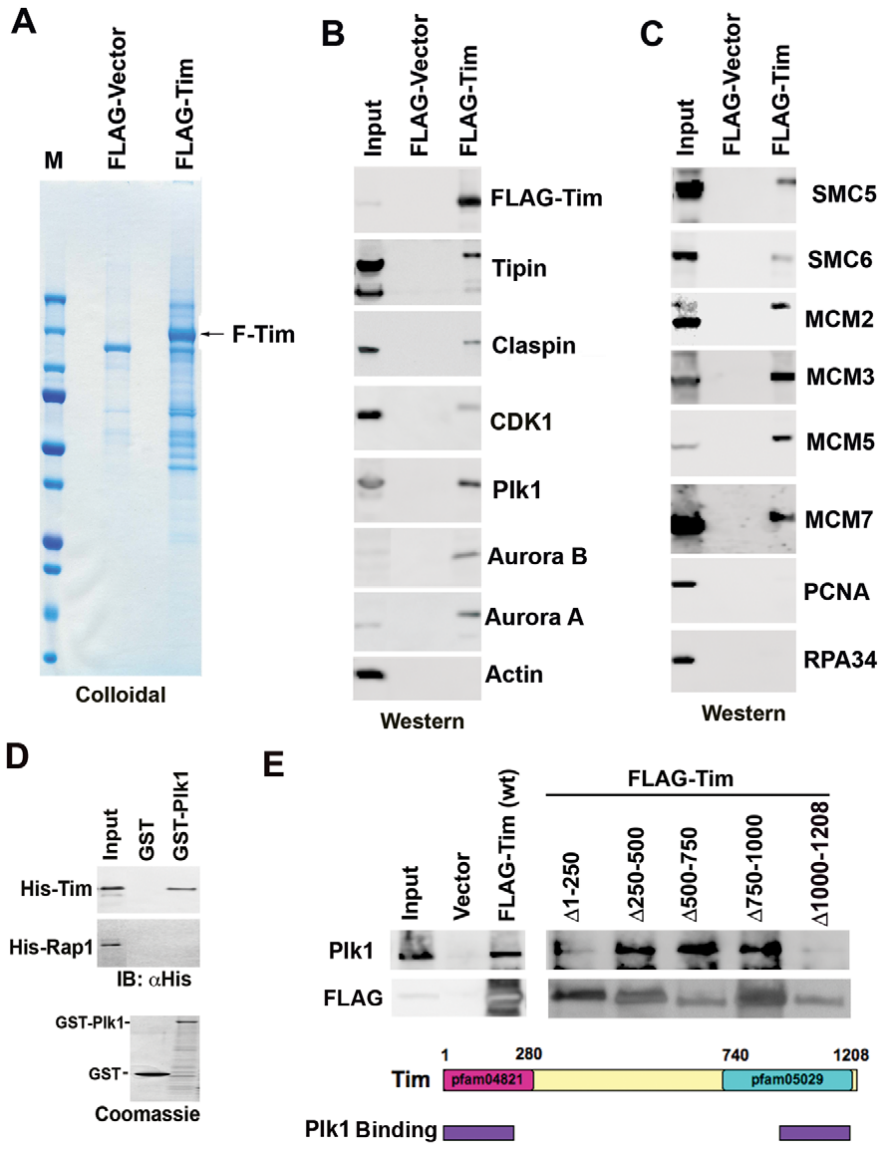
Proteomic analysis of FLAG-Tim complex. A) FLAG-Tim expressing 293 cell lines was used to purify Tim complex using FLAG-agarose. FLAG-vector control or FLAG-Tim proteins were visualized by SDS-PAGE and colloidal blue staining. B) Western blot analysis of FLAG purified proteins from FLAG-Vector or FLAG-Tim expressing cells. Input represents 5% of the starting material from the FLAG-Tim stable cell line. Antibodies for FLAG, Tipin, Claspin, CDK1, PLK1, Aurora B1, Aurora A1, or Actin are indicated. C) Same as in B, except with antibodies to SMC5, SMC6, MCM2, MCM3, MCM5, MCM7, PCNA, and RPA34. D) Baculovirus expressed His-Tim or His-Rap1 were assayed for binding to purified GST or GST-Plk1 by GST-pull down assay. Input and bound proteins were analyzed by Western immunoblot (IB) with anti-His antibody. GST-fusion proteins were visualized by Coomassie staining of SDS-PAGE gels (lower panel). E) Western blot of immunoprecipitates with cells transfected with FLAG-vector, FLAG-Tim wt, or FLAG-Tim deletion mutants (as indicated above each lane). Input is indicated. IPs were assayed by Western blot for Plk1 (top panel) or FLAG (lower panel). Schematic of Tim protein showing that amino and carboxy-terminal conserved domains as pfam04821 and pfam05029, and summary of Plk1 binding is indicated with purple rectangles.

To investigate the molecular mechanisms through which Tim regulates replication termination and cell cycle progression, we generated a stable human cell line expressing FLAG-tagged Tim protein. FLAG-Tim protein was affinity purified and compared to a FLAG-vector control cell line for proteins that associate specifically with Tim (Fig. 1A). Polypeptides enriched in Flag-Tim samples were excised and analyzed by LC/MS/MS. Among the most abundant polypeptides were Tim, Tipin, and Claspin, which was expected from previous studies with mammalian, frog, and yeast models of Tim (Fig. 1B). We also identified several mitotic entry kinases, including CDK1, Plk1, Aurora A and Aurora B as Tim-associated proteins and validated the specific binding to Tim by Western blot analysis (Fig. 1B). In addition, several subunits of the replicative helicase (MCM2, 3, 5, and 7), and the structural maintenance of chromosome proteins (SMC5 and 6) were identified among the Tim-associated proteins by LC/MS/MS and validated by Western blot (Fig. 1C). Other replication proteins, including PCNA and RPA34, which had been shown to interact with Tim in other studies [45], were not found in our Tim-associated complex, suggesting that Tim may form multiple and distinct protein complexes (Fig. 1C). Interactions between endogenous Tim and Plk1 protein was demonstrated by co-immunoprecipitation from Raji nuclear extracts (Fig. S1). Furthermore, the interaction between Tim and Plk1 was insensitive to Dnase I treatment, indicating that the binding was not mediated by non-specific DNA or chromatin linkages (Fig. S1).

To determine if Tim could interact directly with one of the mitotic kinases, we purified His-tagged Tim protein from baculovirus and GST-tagged Plk1 from E. coli. We compared the ability of GST-Plk1 or GST to bind His-Tim or a control protein His-Rap1 using a GST-pull down assay. We found that GST-Plk1, but not GST alone could efficiently pull down His-Tim, but not His-Rap1 (Fig. 1D, top panel). This suggests that Tim and Plk1 polypeptides can interact independently of other cellular proteins.

The Tim protein contains two highly conserved domains spanning the amino terminus (aa 1–270) and the carboxy-terminus (aa 750–1208). To determine if these domains were important for interaction with Plk1, we generated a series of deletion mutations in Tim. Tim deletion mutants were expressed as FLAG-tagged proteins in 293 cells and assayed for their ability to coIP with endogenous Plk1 (Fig. 1E). We found that Tim deletions Δ1–250 and Δ1000–1208 reduced their capacity to CoIP with Plk1. Quantification of multiple IP experiments and quantification by densitometry indicate that the Tim amino terminal domain (Δ1–250) is largely responsible for Plk1 interaction (Fig. S2A). Interestingly, FLAG-Tim (Δ1–250) and Tim (Δ250–500) transfected cells had altered cell cycle profiles, suggesting the Tim amino terminal region is important for both Plk1 binding and proper execution of cell cycle events (Fig. S2B).

### Mitotic colocalization of Tim and Plk1

**Figure 2:**
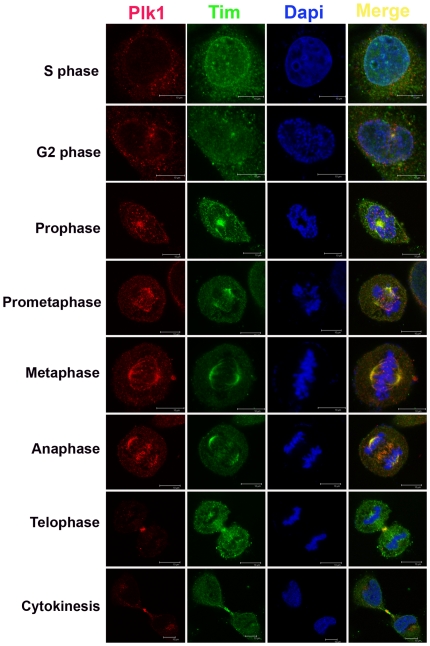
Colocalization of Tim and Plk1 during G2 and M phases. HeLa cells were synchronized by double thymidine block and release, and then assayed by indirect immunofluorescences with antibodies to Plk1 (red), and Tim (green). DNA is stained with Dapi (blue) and merge images are shown in the rightmost panel. Cell cycle stages are indicated to the left of each image.

To determine if Tim colocalized with Plk1 *in vivo*, we used confocal microscopy and indirect immunofluorescence (IF) to analyze the subcellular localization of endogenous Tim and Plk1 in HeLa cells (Fig. 2). Since Plk1 has well-established roles in mitosis, we examined colocalization between Plk1 and Tim throughout the mitotic cell cycle. In S phase, the nuclear staining of Tim and Plk1 are mostly diffuse, partially colocalized, with some accumulation at the nuclear periphery. In G2 cells, Plk1 and Tim concentrate at the centrosomes. In prophase, Plk1 and Tim localize to the chromosome congregation centers. In prometaphase and metaphase cells, Tim and Plk1 concentrate at the centrosomes, as well as along the microtubule network linking centrosomes to chromatin. In anaphase, Tim and Plk1 colocalize strongly at the mid-body, but this colocalization changes into discrete compartments by telophase and cytokinesis. A similar pattern of colocalization was also observed between Tim and Aurora A (Fig. S3). Tim antibody specificity was confirmed by Western blotting and by IF experiments with Tim-targeted siRNA depleted cells (Fig. S4). These data indicate that Tim partially colocalizes with Plk1 and Aurora A at multiple stages and subcellular structures during G2 and mitosis.

### Tim depletion causes metaphase chromosome aberrations and mitotic catastrophe

**Figure 3:**
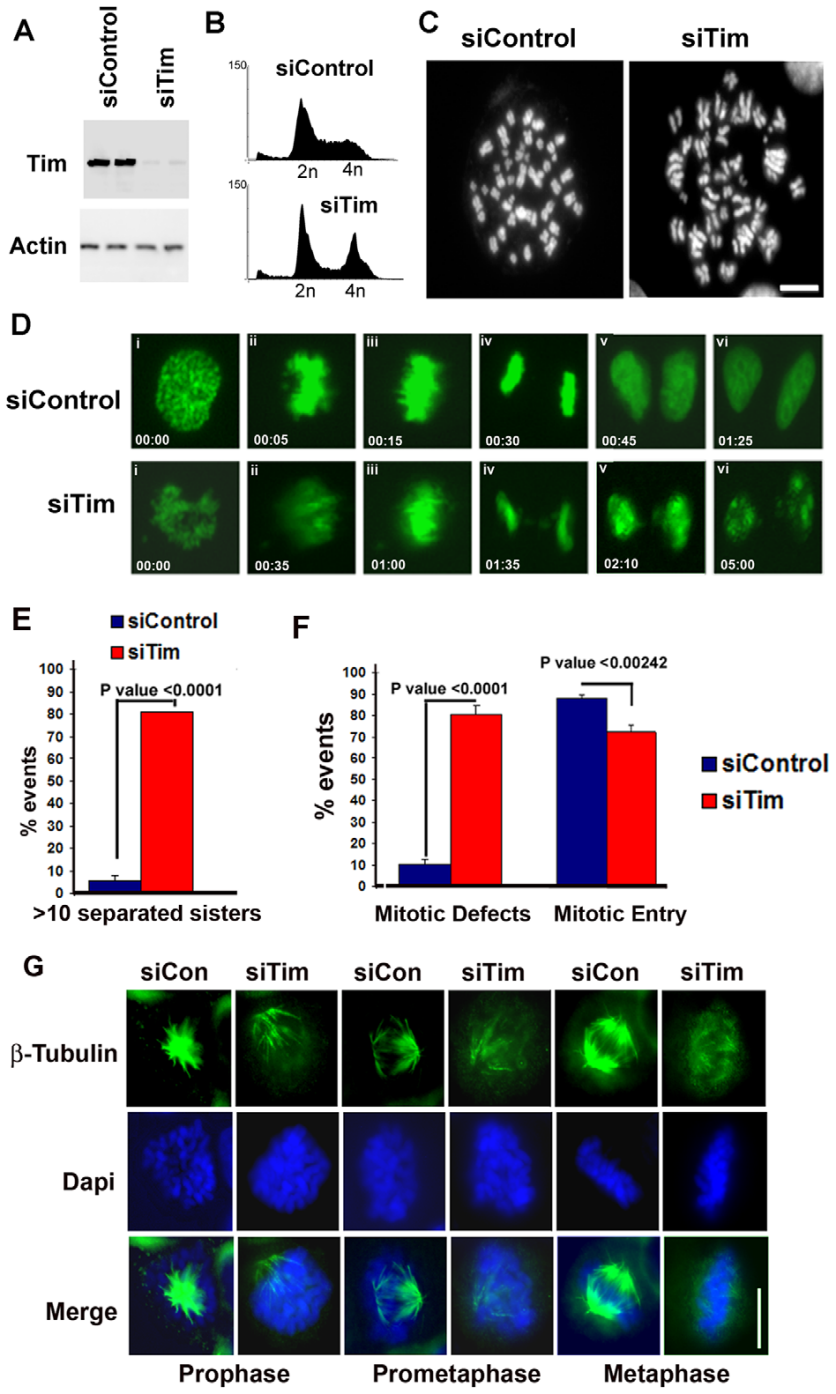
Timeless depletion causes mitotic chromosome defects. A) HCT116 cells were transfected in duplicate with siControl or siTim and then assayed by Western blot at 24 hrs post transfection. Total cell extracts were probed with antibodies to Tim (top panel) or actin (lower panel). B) Cell cycle profiles of siControl or siTim transected HCT116 cells were generated by FACS analysis after propidium iodide staining. C) Metaphase spreads of siControl or siTim transfected HCT116 cells were generated at 24 hrs post-transfection. D) Images from time-lapsed micrographs of mitotic cell stages in siControl or siTim transfected HeLa cells that stabely express GFP-H2B to mark chromosomes. i) Prophase, ii) Prometaphase, iii) Metaphase, iv) Anaphase, v) Telophase, vi) Cytokinesis. E) Quantification of cells containing >10 separated sisters in metaphase spreads as represented in panel C (n = 86cells). Error bars represent standard deviation from the mean, and P values are derived from Chi-square analysis. F) Quantification of the number of mitotic defects (lagging chromosomes, failure to segregate, failure to progress to anaphase, failure during cytokinesis) observed for 5 independent movies (siControl, n = 207cells, siTim, n = 203 cells). Error bars represent standard deviation from the mean, and P values are derived from Chi-square analysis. G) Microtubule organization and centrosome disorganization in Tim depleted HeLa cells. Metaphase cells were stained for tubulin (green) by indirect immunofluorescence and DNA with Dapi (blue).

The function of Tim in cell cycle control and chromosome maintenance was investigated through siRNA and shRNA depletion. Two different siRNA and a tet-inducible shRNA stable cell line were examined for the effects of Timeless depletion in human cell lines. siRNA depletion of Tim in HCT116 cells caused >90% depletion of total Tim protein, as measured by Western blot (Fig. 3A). The effect of siRNA depletion of Tim was first examined by cell cycle profile using FACS analysis of propidium iodide stained cells, 24 hrs post-transfection (Fig. 3B). We found that siTim depleted cells accumulated in G2/M with a corresponding reduction in S and G1 cells. Live cell imaging using GFP-tagged histone H2B cells revealed that Tim transfected cells were able to enter mitosis, but could not complete a normal M phase (Movie S1 and S2). Analysis of live cell images revealed several defects during mitosis, including chromosome condensation defects and formation of lagging chromosome structures (Fig. 3D and F). To further assess the defects during M phase, we examined metaphase chromosomes after colcemid treatment (Fig. 3C). Examination of metaphase spreads revealed a striking reduction (∼20 fold) in sister chromatid cohesion (Fig. 3E). The failure to generate sister chromatid cohesion was also observed in a stable 293 cell-line expressing an inducible shRNA that efficiently depletes Tim (Fig. S5). This is consistent with findings from Leman et al. showing that chromosome cohesion is compromised in Tim depleted cells [46]. Bipolar spindle formation and microtubule assembly on condensed chromosomes were also disorganized in siTim-depleted cells (Fig. 3G). Although chromatin condensed during metaphase, the formation of stable microtubules emerging from two opposing poles was largely absent after Tim depletion (Table S1). Taken together, these data indicate that Tim is required, directly or indirectly, for multiple mitotic events, including chromosome condensation, sister chromatid cohesion, bipolar microtubule organization, and microtubule assembly on condensed chromatin.

### Tim Depletion Alters Mitotic Entry Kinase Stability

**Figure 4:**
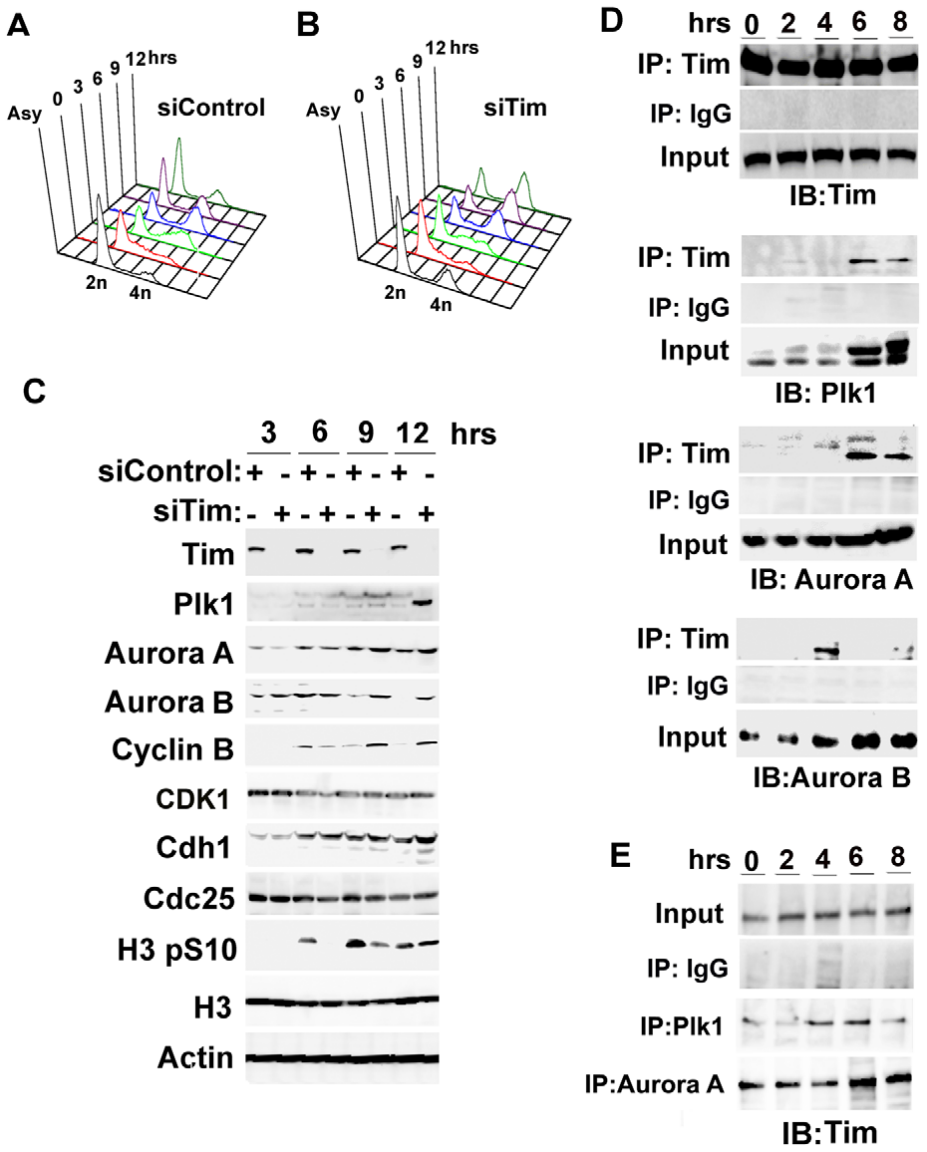
Cell cycle dependent interactions between Tim and mitotic entry kinases. A–B) siControl and siTim transfected HCT116 cells were synchronized by double thymidine and then assayed by PI staining and FACS analysis for cell cycle profile. C) Western blot of total cell extracts of synchronize HCT116 cells after siControl or siTim at cell stages shown in panels A and B for 3, 6, 9, and 12 hrs post-release from thymidine block. Western blot with antibody to Tim, Plk1, Aurora A1, Aurora B1, Cyclin B1, CDK1, Cdh1, Cdc25, histone H3 phospho S10, hitone H3, or Actin, are indicated. D) Immunoprecipitation with anti-Tim or control IgG antibody with extracts from HCT116 cells at 0, 2, 4, 6, or 8 hrs post-arrest from thymidine block. IPs were assayed by Western immunoblot (IB) with Tim, Plk1 Aurora A, or Aurora B antibody as indicated. E) HCT116 cells were synchronized as in panel D, and extracts were subject to IP with antibody to Plk1 (middle panel) or Aurora A (lower panel), followed by Western blot with anti-Tim. Input is shown in top panel, as indicated.

The interaction of Tim with mitotic entry kinases provoked us to examine the effect of siTim depletion on the cell cycle behavior of these proteins. siControl or siTim depleted HCT116 cells were synchronized in G1 by double thymidine block and release. The cell cycle profile of these cells indicated that siControl cells progressed into S phase by 3 hrs, and G2/M by 6 hrs, and G1 reappearing by 9 and 12 hrs (Fig. 4A). siTim depleted cells had a similar cell cycle profile, with the exception that many fewer cells exit M phase after 9 and 12 hrs (Fig. 4B). Protein levels for several cell cycle regulated proteins were examined by Western blot at 3, 6, 9, and 12 hrs after release (Fig. 4C). As expected, Western blot with anti-Tim revealed a >90% reduction in Tim protein in siTim transfected cells. Remarkably, we found a significant elevation of Plk1, Aurora A and Aurora B, as well as Cyclin B1, at 9 and 12 hrs post-release in the siTim transfected cells. As expected, CDK1 protein levels did not change. This abnormal accumulation in cyclin B1 and mitotic entry kinases in siTim treated cells is consistent with a failure to complete M phase, which occurs in siControl cells by 9 hrs post-release. We also observed that histone H3 S10 phosphorylation, which is a mark for mitotic cells, was significantly delayed in siTim treated cells relative to siControl. Total histone H3 levels and other cell cycle regulatory proteins (CDH1, Cdc25) were indistinguishable in the siControl compared to the siTim depleted cells. Tim depletion did not lead to the activation of the DNA damage checkpoint kinase Chk1, and only activated Chk2 kinase at 12 hrs post-release from double-thymidine block, reflecting a failure to exit mitosis (Fig. S6A). Tim depletion did not activate mitotic checkpoint protein BubR1, and prevented the normal activation of BubR1 after nocodazole treatment [47] (Fig. S6B). These findings indicate that Tim depletion does not induce an intra-S phase or mitotic checkpoint response in HCT116 cells. Rather, Tim depletion leads to an aberrant stabilization of the mitotic kinases Plk1, Aurora A and B in M phase, and a delay in M phase-associated histone H3 S10 phosphorylation.

### Cell cycle-dependent interaction of Tim with mitotic kinases

To determine if the interaction between Tim and mitotic kinases was cell cycle dependent, we examined the interactions at various stages of the cell cycle (Fig. 4D). HCT116 cells were synchronized by double-thymidine block and release and monitored by FACS essentially as shown in panels 4A and B. Synchronized cells were then subject to immunoprecipitation (IP) with anti-Tim or control IgG. IPs were first assayed by Western blot with anti-Tim antibody to show that Tim was precipitated equally at all stages of the cell cycle (top panels). Western blot with antibodies to Plk1, Aurora A, or Aurora B revealed that these mitotic entry kinases associate with Tim preferentially in G2 and M phases, with Aurora A and Plk1 interactions peaking at 6 hrs, and Aurora B interactions peaking at 4 hrs after double thymidine release (Fig. 4D). The increase interaction between Tim and Plk1 or Tim and Aurora A corresponded to the increase in abundance of these proteins as cells progressed through the cell cycle. Cell cycle association of Tim was also observed in the reverse IP with either Plk1 or Aurora A (Fig. 4E). We conclude that Tim interacts with these mitotic entry kinases predominantly at G2 and M phases of the cell cycle, consistent with findings from our immunolocalization studies (Fig. 2 and S3).

### Tim is required for mitotic kinase activity

**Figure 5:**
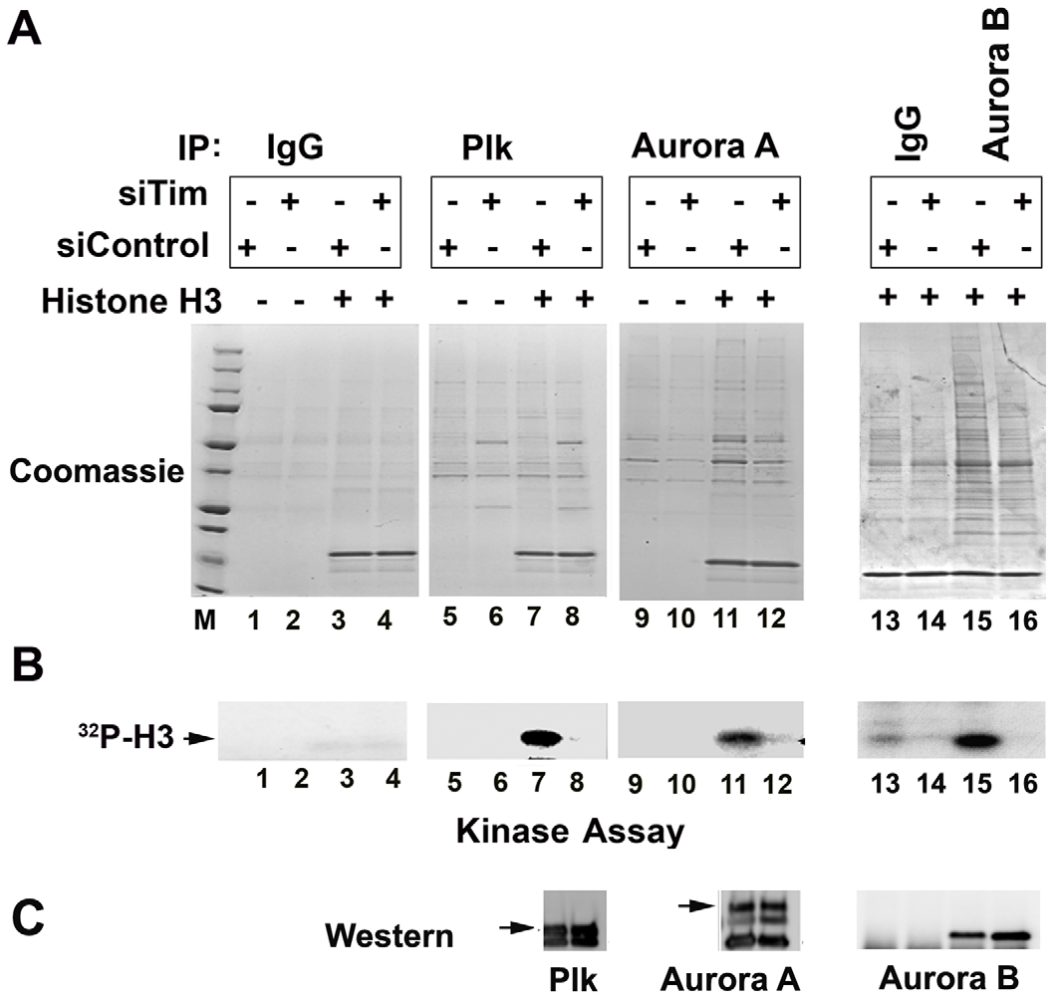
Tim is required for Plk1 and Aurora A kinase activity in vitro. A–B) HCT116 cells were transfected with siControl (odd lanes) or siTim (even lanes) and then subject to IP with IgG (lanes 1–4, and 13, and 14), Plk1 (lanes 5–8), Aurora A (lanes 9–12), or Aurora B (lanes 15 and 16). Purified histone H3 substrated was added to lanes 3,4, 7,8, 11, 12, 13–16, and the IPs were incubated with ^32^P-γATP under kinase conditions for 30 min. Coomassie blue stain of SDS-PAGE containg the kinase reaction is shown in panel A. The autoradiogram of the ^32^P-histone H3 is shown in panel B and indicated by the arrow. C) Western blot of IP material used for kinase reactions for Plk1, Aurora A, and Aurora B, as indicated.

Experiments shown in Figure 4C suggest that Tim is required for the timely phosphorylation of histone H3 S10 during G2/M phase (6 hrs post release). Previous studies have implicated Aurora B kinase in phosphorylation of histone H3 S10, and shown that several mitotic kinases, including Aurora A, can phosphorylate histone H3 *in vitro* [38], [39]. To determine if Tim is required for the Plk1, Aurora A, or Aurora B kinase activity, we assayed immunoprecipitates of these proteins for kinase activity *in vitro* using histone H3 as a substrate (Fig. 5). HCT116 cells were transfected with siControl or siTim, synchronized by double thymidine block as shown in Fig. 4A and B. Cells synchronized in G2/M (6 hrs) were then subject to immunoprecipitation with IgG, Plk1, Aurora A, or Aurora B. The IPs were then tested for *in vitro* kinase activity without or with exogenous histone H3 substrate added to the reaction. We found that IPs of Plk1, Aurora A, and Aurora B from Tim depleted cells had substantially less kinase activity than IPs from siControl treated cells (Fig. 5B). Coomassie blue staining of the IPs and kinase reactants indicate that similar amounts of IP and substrate were included in each reaction (Fig. 5A) and Western blot of the IPs show that identical amounts of Plk1, Aurora A, and Aurora B proteins were recovered from siControl and siTim IPs and present in each reaction (Fig. 5C). Based on these findings, we conclude that Tim is required for G2/M activation of Plk1, Aurora A and Aurora B kinase activity, as measured by phosphorylation of histone H3 *in vitro*.

### Tim is required for activation of Plk in vivo

**Figure 6:**
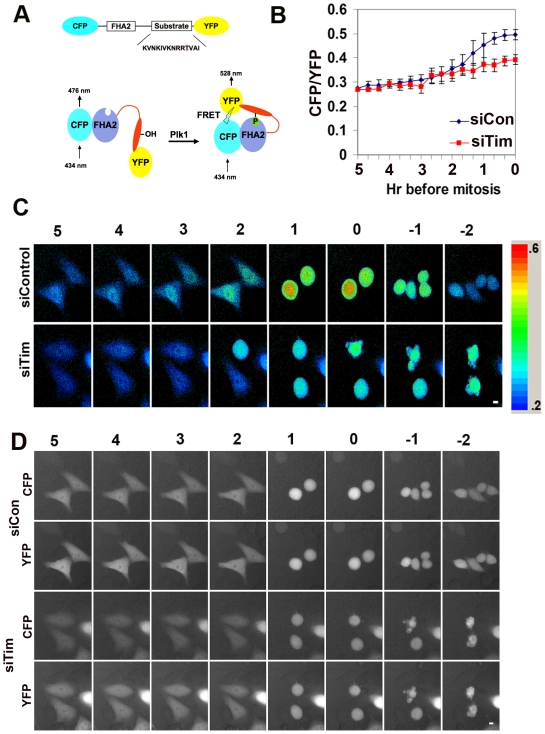
Tim depletion compromises Plk activity in vivo. A) Schematic of the Plk1 acceptor substrate used for FRET and affect of phosphorylation on protein conformation as depicted by Violin et al. [65]. B) Quantification of the average and standard deviation of CFP/YFP emission ratio in presence of siTim (red line n = 9cells) or siControl (blue line n = 8cells). C) Time-lapse sequence showing the false-coloured CFP/YFP ratio of a Hela cells expressing a FRET probe for Plk1 activity. Top panel, siControl;lower panel siTim transfection. Time related to mitosis is indicated above the image (hours). D) Representative example of YFP and CFP channel images from siControl and siTim transfected cells used for FRET analysis in Figure 5C.

To determine whether Tim is required for mitotic kinase activation *in vivo*, we utilized an established FRET-based assay with a substrate containing a consensus acceptor site for Plk [44]. This FRET substrate has been characterized in several other studies and been shown to be highly selective for Plk family of kinases during the pre- and early phase of mitosis [44]. We therefore tested whether Plk activity was altered in cells transfected with siTim relative to siControl (Fig. 6). Transfected cells were visualized by live cell imaging combined with FRET analysis. Quantification of FRET signal revealed that Plk activity was significantly reduced (>50%) in siTim transfected cells relative to siControl. Plk activity increased significantly at ∼1 hr prior to mitotic cell condensation in siControl cells, but failed to reach similar levels in siTim transfected cells. These findings indicate that Tim depletion partially blocks the activation of Plk family kinases during the G2 and early M stages of mitosis *in vivo*.

### Tim is required for Plk1 and Aurora A association with centromere DNA

**Figure 7:**
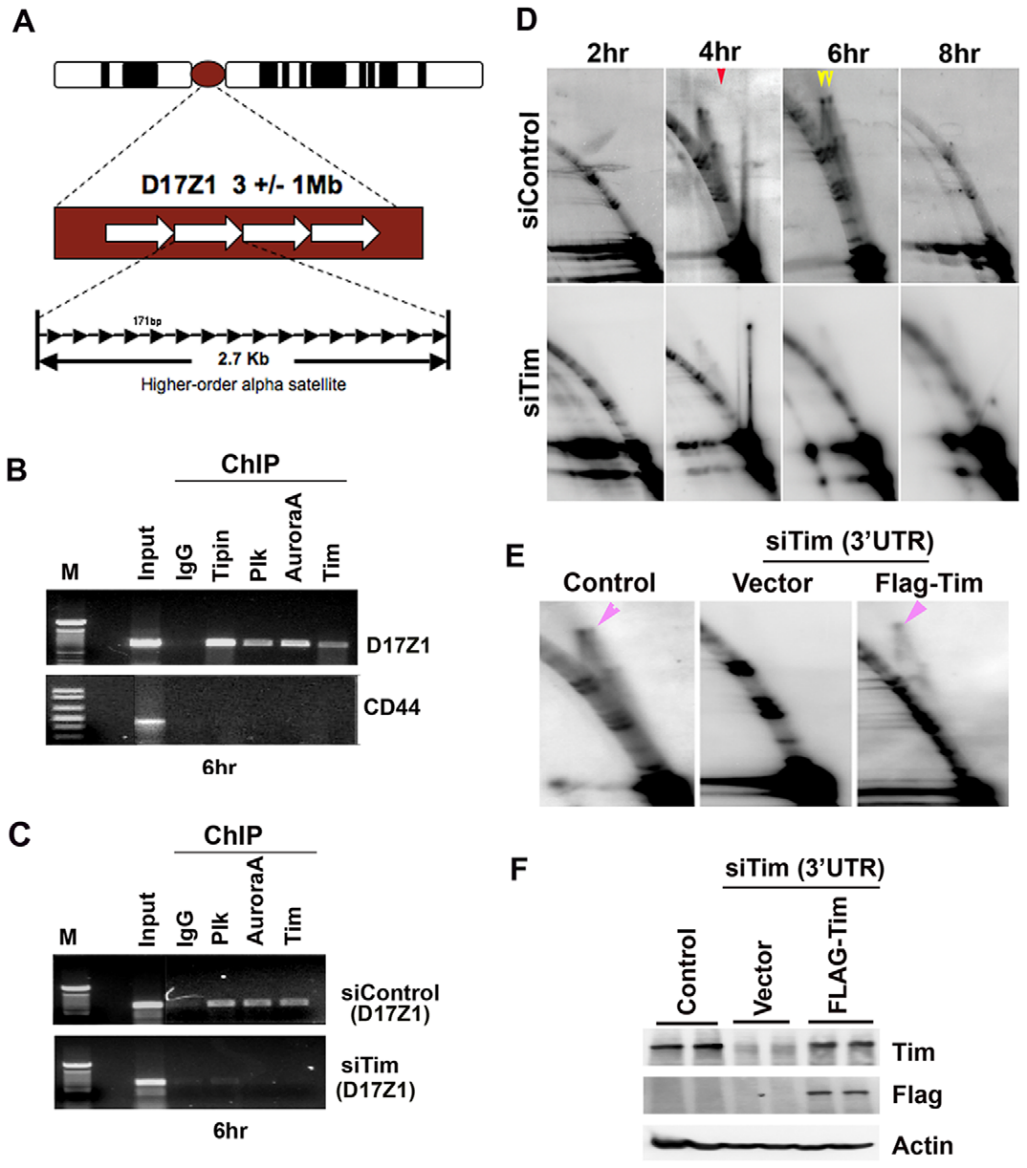
Centromere replication fork stability and mitotic kinase association are Tim-dependent. A) Graphic representation of the higher-order alpha-satellite repeats found in all human chromosomes. The D17Z1 contromeric probe from chromosome 17 was used for analysis of replication fork and ChIP assays. B) HCT116 cells were synchronized at 6 hrs post-release from double thymidine block. Synchronized HCT116 cells were subject to ChIP with antibodies to Tipin, Plk1, Aurora A1, Tim, or control IgG as indicated above each lane. Input represents 1∶100 dilution of starting material. D17Z1 centromere is shown in the top panel, and control CD44 gene is shown in lower panel. M represents 100 bp ladder. C) HCT116 cells were transfected with siControl I (top panel) or siTim (lower panel), synchronized by double thymidine block and release, and the subject to ChIP assay at 6 hr post-release. Antibodies to Plk1, Aurora A1, Tim, or control IgG are indicated above each lane. ChIP DNA was assayed for centromere D17Z1 region. D) Two dimensional neutral agarose gel electrophoresis and Southern blot analysis of the D17Z1 centromere alpha-satellite repeat region. 2D gels were analyzed for siControl transfected HCT116 cells synchronized at 2, 4, 6,or 8 hrs post-release from double thymidine block (top panels), or for siTim transfected cells isolated at 4 and 6 hr post-release. Red arrow in siControl 4 hrs indicates the replication origin bubble arc. Yellow arrows in siControl 6 hrs indicate the replication pause and termination structures. E) HCT116 cells transfected as in panel C were assayed by 2D neutral agarose gel electrophoresis and Southern blot with probe for the D17Z1 satellite repeat. DNA recombination structures (X-structures) are indicated by the pink arrowheads. F) Western blot analysis of HCT116 cells transfected with control siRNA or siRNA targeting Tim 3′UTR, as indicated above. 3′UTR targeted cells were then transfected with either control vector or FLAG-Tim expression plasmids. Western blots for Tim, FLAG, or Actin are indicated.

Plk1 has been implicated in the direct binding and processing of the kinetochore structures that form around centromere DNA [28]. Aurora A localizes primarily to the centrosome [48] but studies in budding yeast suggest that centrosomes are linked to centromere DNA during G2/M transition [49]. To explore the possibility that Tim, Plk1, and Aurora A may converge at centromeric DNA in G2, we examined human centromeric DNA by chromatin immunoprecipitation (ChIP) assays for interaction with these proteins (Fig. 7A–C). All the centromeres of human chromosomes contain an array of higher order alpha-satellite repeats (Fig. 7A) [40]. We assayed the ability of Tim, Tipin, Plk1, and Aurora A to interact with the chromosome 17 centromere alpha satellite repeat DNA (D17Z1) which can be uniquely amplified by PCR (Fig. 7A). We found that Plk1 and Aurora A, along with Tim and Tipin interact with the D17Z1 centromere DNA in HCT116 cells synchronized at G2/M (6 hrs as shown in Fig. 4A). None of these proteins interact with control DNA from the CD44 gene (Fig. 7B). When Tim was depleted by siRNA transfection of HCT116 cells, neither Plk1 nor Aurora A associated with centromere repeat DNA (D17Z1) (Fig. 7C, lower panel). These findings suggest that Tim, Tipin, Plk1 and Aurora A can colocalize at centromere repeat DNA in G2, and that Tim is required for this colocalization.

### Tim is required for Plk binding to centromere DNA and formation of replication termination structures

Centromere function is also known to depend on the formation of replication termination and recombination-like structures that promote sister-chromatid cohesion [40]. We therefore examined the role of Tim in promoting recombinational structures at centromeres, and whether this correlated with the recruitment of Plk1 and Aurora A to centromeric DNA (Fig. 7D). We assayed the replication fork pausing structures at the chromosome 17 centromere by two dimensional neutral agarose gel electrophoresis. DNA was isolated using CTAB, a cationic detergent that preserves Holliday junctions and other recombination-like structures formed during DNA replication [50]. We found that the alpha-satellite repeats within chromosome 17 centromere form a vertical spike, indicative of an X-structure (yellow arrows), as well as a weaker bubble arc (red arrow), reflective of an origin bubble in S phase (4 hrs post release from double thymidine block) (Fig. 7D, top panel). By G2/M phase (6 hrs post-release) the major structures include two vertical spikes reflective of an X-structure or replication termination site (Fig. 7D, top panels). In siTim transfected cells, no bubble arc could be detected, and the vertical spikes were diffuse and poorly formed in both S (4 hrs) and G2 (6 hrs) phases of the cell cycle (Fig. 7D, lower panel). Cell cycle stage was confirmed by FACS for both siControl and siTim transfected cells (data not shown). To further validate that Tim was indeed required for the formation of X-structures at centromere repeat DNA, we re-expressed FLAG-Tim in cells where endogenous Tim was depleted by a different siRNA targeting the 3′ UTR of endogenous, but not ectopic Tim (Fig. 7E and F). Western blot confirmed that the 3′ UTR targeting siRNA efficiently depletes Tim, and that FLAG-Tim was expressed at levels comparable to endogenous Tim (Fig. 7F). We also observed that the 3′ UTR targeting siRNA had identical phenotype to the previously utilized Tim siRNAs (Fig. S7). Using this siRNA depletion and reconstitution system, we found that X-structure formation at centromere repeats is strictly dependent upon Tim expression (Fig. 7E). X-structures (purple arrows) were observed efficiently in siControl cells, but not in siTim transfected cells. However, re-introduction of FLAG-Tim restored X-structure formation at the centromere repeat DNA. This indicates that Tim is required for replication fork processing at centromeres, consistent with the well-established role of the yeast orthologues of Tim in regulating replication fork stalling and termination [51].

## Discussion

**Figure 8:**
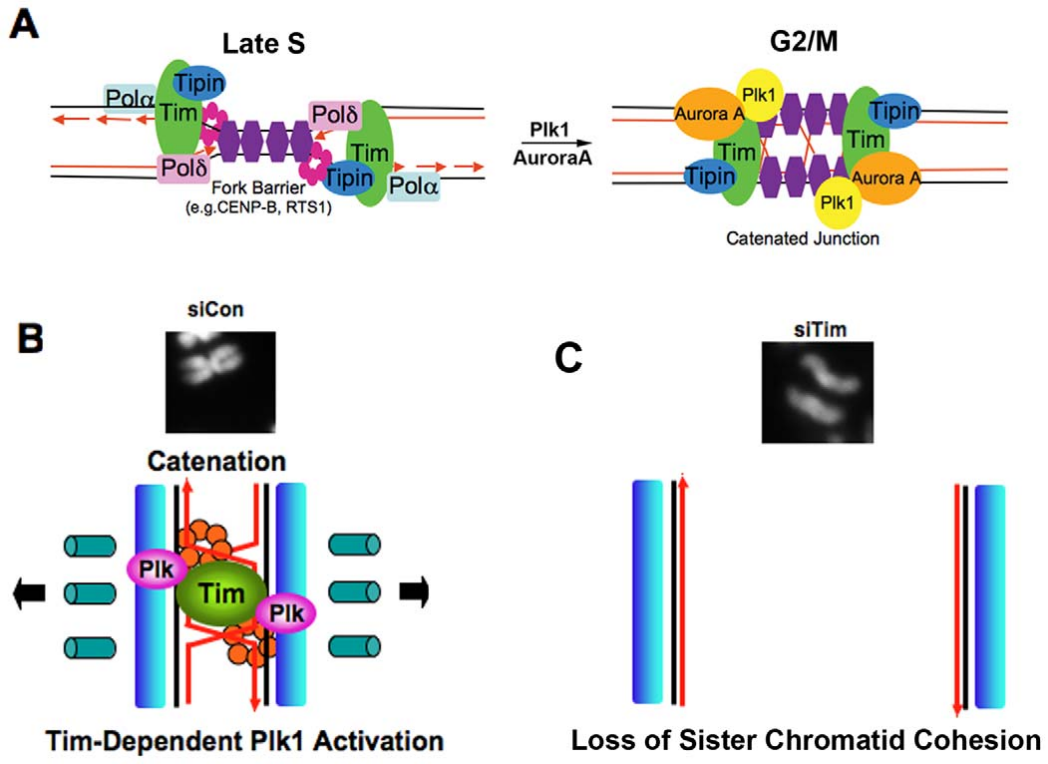
Tim coordinates replication termination with mitotic kinase activation. **A**) Model of Tim coordinating replication termination with mitotic kinase activation. Replication forks approach fork-blocking proteins (blue) in S phase lead to the formation of recombination structures in G2. Tim is required for the formation of the DNA recombination structure and the recruitment of Plk1 and Aurora A, as demonstrated at centromeric DNA. B) Metaphase chromosome cohesion is established through Tim-dependent DNA recombination or termination structures (X-structure), which leads to the activation of mitotic kinases (e.g. Plk1). C) Tim depletion causes a loss of DNA catenation, loss of Plk1 recruitment to centromere repeats, loss of Plk activation, and loss of sister-chromatid cohesion.

In this study, we show that Tim protein could be isolated as a stable complex with mitotic entry kinases Plk1, Aurora A and B, and CDC2, as well as with components of the replicative helicase (MCM subunits) and replication fork monitoring proteins (Claspin and Tipin) (Fig. 1). We found that Tim bound directly to Plk1 protein *in vitro* (Fig. 1D and E) and colocalized with Plk1 during multiple stages of M phase *in vivo* (Fig. 2). Cells depleted of Tim with siRNA or shRNA had defects in metaphase chromosome condensation, sister-chromatid cohesion, centrosome and microtubule organization, and cytokinesis (Fig. 3). Tim depletion caused an abnormal accumulation of Cyclin B1, Plk1, and Aurora A, and a delay in phosphorylate histone H3 S10 in G2/M phase (Fig. 4). Plk1, Aurora A, and Aurora B proteins derived from Tim depleted cells were incapable of phosphorylating purified histone H3 substrate in cell-free reactions (Fig. 5), and were diminished for Plk1 activity *in vivo* as measured by FRET assay (Fig. 6). Finally, we showed that Tim was required for the recruitment of mitotic kinases to centromeric alpha-satellite repeat DNA (Fig. 7B–C), as well as the formation of recombination structures at centromeric DNA (Fig. 7D–F). Taken together, these data suggest that Tim plays a critical role in coordinating mitotic kinase activity with the formation of replication termination structures in G2/M phase of the cell cycle (Fig. 8).

### Tim forms a stable complex with mitotic kinases

Earlier studies have shown that Tim, and its evolutionarily conserved orthologues, interact with several proteins associated with DNA replication fork and intra-S phase checkpoint regulation, including Tipin, Claspin, MCMs, and RPA34 [18], [20], [21], [45]. We isolated Tim as a FLAG-tagged protein from stable 293 cell lines, and identified Tipin, Claspin, and MCMs, as expected, but did not recover detectable RPA34 or PCNA (Fig. 1). Instead, we found that mitotic kinases Plk1, Aurora A and B, and CDK1 were highly enriched in FLAG-Tim affinity purified preparations. The failure to find RPA34, which has been shown to interact with Tipin at single stranded DNA [21], [45], suggests that Tim may form multiple independent complexes and our isolation methods may enrich for a G2/M-specific form of Tim. The interaction between Tim and mitotic kinases was observed predominantly in G2/M cells (Fig. 4), suggesting that Tim may change interaction partners in a cell cycle-dependent manner. This is consistent with the observation that Tim can function at more than one stage of the cell cycle, namely at the replication fork during S phase [20], [52], and in the activation of mitotic kinases in G2. The interaction of Tim with mitotic kinases can be detected in G2 and M phases (Fig. 4D), and therefore may provide a mechanism for regulation of M phase events, including sister-chromatid cohesion [46], [53].

### A Direct Role for Tim in M phase Progression

Tim depletion by siRNA or shRNA had no effect on the normal progression of S phase (Fig. 4A and B). In contrast, Tim depletion prevented the progression and completion of normal M phase events, resulting in defects in microtubule organization, chromosome condensation, sister-chromatid cohesion, and formation of lagging chromosomes (Fig. 3). While some of these events may be due to replication errors resulting from the generation of single stranded DNA, they were not sufficient to cause an arrest in S phase, nor an activation of intra-S phase checkpoint kinases Chk1 and Chk2 (Fig. S6). The activation of Chk1 was detected in siTim transfected cells, but only at 12 hrs after S phase, when control cells have exited mitosis and re-entered G1. Thus, it is not likely that intra-S phase checkpoint activation mechanisms can account for the many mitotic aberrations observed after Tim depletion. Similar defects were observed with multiple siRNA (Fig. S7) and inducible shRNA (Fig. S5), as well as by overexpression of Tim truncation mutants (Fig. S2), indicating that these were not off-target effects of the siRNAs. The physical interaction of Tim with Plk1 and Aurora kinases in G2/M phase and colocalization of Tim at mitotic structures, including centrosomes and midbody (Fig. 2 and S3), suggest that Tim has additional functions outside of its well-established role in replication fork monitoring. Multifunctional properties have been described for numerous other proteins, including replication factors like ORC6, which is a component of the origin recognition complex as well as a functional component of the centrosome [54]. Plk1 has also been shown to function at multiple locations and stages of the cell cycle, including regulation of replication factors [55]. Our data suggests that Tim functions at replication termination structures, as well as in subsequent mitotic events in close association with mitotic kinases.

### Tim is required for mitotic kinase function

Aurora A and Plk1, along with CDC2, regulate multiple events important for entry into and progression through mitosis [28], [55], [56]. Aurora A is thought to be required for Plk1 activation, and both proteins require CDC2 activity for M phase progression [8]. Both Aurora and Plk1 kinases have multiple substrates and it is not yet known which substrates are essential for mitotic cell progression. H3 S10 phosphorylation has been used as a mark for mitotic cells, and the Aurora B kinase has been implicated in this process [39]. Our data indicates that mitotic phosphorylation of H3 S10 is partly dependent on Tim (Fig. 4 and 5). We found that Tim depletion caused a delay and reduction in total cellular H3 S10 phosphorylation (Fig. 4C). Using histone H3 as an *in vitro* substrate, we found that Plk1 and Aurora IP-kinase activity was severely compromised in siTim depleted cells (Fig. 5). FRET assays also indicate that Plk1 has reduced activity in Tim siRNA depleted cells *in vivo* (Fig. 6). While histone H3 may not be the physiological substrate of Plk1 and Aurora A *in vivo*, our finding are consistent with a delay and decrease of Aurora A and Plk1 mitotic kinase activity in Tim depleted cells. These findings strongly suggest that Tim is required for the timely activation of Plk1 and Aurora A kinases in G2 and M phase of the cell cycle.

### Recruitment of mitotic entry kinases to centromeres

Aurora A has been implicated in the G2 stage activation of Plk1, but it is not completely clear what triggers this event [44], [56]. We found that Aurora A, Plk1, Tim, and Tipin could colocalize by ChIP at the alpha-satellite repeat DNA in the chromosome 17 centromere (Fig. 7B). This interaction was observed in cells synchronized in G2 and was abolished by Tim depletion (Fig. 7C). This finding is consistent with subcellular colocalization (Fig. 2 and S3), and IP experiments (Fig. 4D), demonstrating a cell cycle-dependent interaction of Tim with Aurora A and Plk1. While Aurora A is typically thought to localize to centrosomes and regulate bipolar spindle formation in M phase, its location and function in G2 has not been clearly defined. Studies in budding yeast suggest that centromeres may transiently interact with centrosomes at the nuclear envelope as chromosomes are prepared for entry into mitosis [49]. Our finding suggest that Aurora A colocalizes with Plk1 at centromere repeats in G2, when DNA replication is terminated and recombination structures are formed. These events are likely to occur prior to the complete segregation of Aurora A at the centrosomes, and is consistent with a nuclear function of Aurora A in the G2 activation of Plk1.

### Tim is required for replication termination structures at centromere repeat DNA

DNA recombination structures were observed at the human centromere alpha satellite repeats in G2 phase (Fig. 7D). In Tim depleted cells, these recombinational X-structures were detected at very low levels relative to Y-arcs at G2 and M phase (Fig. 7D). Furthermore, restoration of Tim in siTim cells rescued the formation of X-structures, indicating that this effect is Tim-dependent (Fig. 7E). The weak formation of X-structures in siTim depleted cells indicates that these cells are at similar stages in the cell cycle, but fail to generate robust DNA recombination structures at sites where Tim normally localizes and recruits Plk1 and Aurora A (e.g. centromeric alpha satellite repeats). Furthermore, FACS analysis indicated that these cells were in similar stages of the cell cycle when analyzed for DNA structure (Fig. 7D–E) and ChIP analysis (Fig. 7B and C). While we can not formally rule out the possibility that Tim depletion induces some level of S phase checkpoint activation that blocks or delays recombination structure formation, our findings are most consistent with a required role of Tim in generating recombination structures at replication fork barriers [18]. The yeast orthologue of Tim, Swi1, is required for stabilizing replication forks at some DNA polymerase pause sites, like those found at the mating type switch locus (Rts1) and centromeres, where replication terminates [11], [12], [57]. These termination sites have also been shown to form recombination-like structures that promote DNA catenation and sister chromatid cohesion. This may partly account for the role of Tim in promoting sister-chromatid cohesion. Recombination structure formation and sister-chromatid cohesion at repeat DNA is also known to involve SMC5 and SMC6 [58], [59], [60]. We found that SMC5 and 6 were both enriched in the FLAG-Tim protein complex (Fig. 1C), and that Tim, Tipin, Plk1, SMC5 and SMC6 associate with centromere and subtelomere repeat DNA in late S phase (data not shown). Thus, mammalian Tim is likely to perform a related function to its yeast orthologues in mediating replication termination at repetitive DNA (e.g centromeres and telomeres), as well as promote sister-chromatin cohesion through formation of catenated DNA.

### Does Tim coordinate mitotic progression with replication termination and circadian clock?

Mammalian Tim has been best characterized for its function in monitoring replication fork stability in S phase, but other functions of Tim can not easily be explained by its intra-S phase functions alone. Our findings indicate that Tim is required for progression through M phase, and we provide evidence that Tim physically and functionally interacts with mitotic kinases in G2 and M. We propose that Tim plays a central role in coordinating replication termination with the early stages of mitotic kinase activation (Fig. 8). The precise biochemical mechanism through which Tim promotes mitotic kinase activation is not yet known, but DNA structural perturbations associated with replication termination and recombination may provoke these signals. DNA structural changes have been implicated in the regulation of *Drosophila* Timeless in response to circadian photosensitivity [61], [62]. While mammalian Tim has diverged significantly from diptera, there is evidence that mammalian Tim may also function in circadian rhythm regulation [52], [63]. Remarkably, the mammalian circadian clock has been shown to regulate mitotic progression through the activation of CDK1 kinase activity and Wee1 gene expression [64]. Whether the regulation of mitotic events described in this study play a role circadian rhythm control, remains an intriguing, but unanswered question. The mitotic defects induced by Tim depletion, including the loss of sister-chromatid cohesion, the lack of bipolar spindle formation, and the lack of mitotic kinase activity, can be explained best by a direct role of Tim in mitotic progression. Whether a single biochemical activity accounts for the S phase and M phase functions of Tim will be an important question for future investigation.

## Supporting Information

Figure S1 **Co-immunoprecipitation of endogenous Tim with mitotic kinases.** A) Asynchronous Raji cell nuclear extracts were subject to immunoprecipitation with either αTim or control IgG antibodies and then treated with 100 µg/ml DNAse I for 30 min (+) or buffer lacking DNase I (−), followed by extensive washing. Eluted proteins were then analyzed by Western blot αPlk1 (panel A) or αAurora A antibodies (panel B).
(TIF)

Figure S2 **Characterization of FLAG-Tim deletion mutants.** A) Quantification of at least three independnt CoIPs for Plk1 after FLAG-IP with extracts from cells transfected with FLAG vector, FLAG-Tim wt, or FLAG-Tim deletion mutants (as indicated and represented by Figure 1E). IP values were quantified as percentage of input for each Tim deletion mutant. B) Tim wt and Tim mutants were transfected and assayed for their dominant negative effects on cell cycle profile using FACS analysis of propidium iodide stained cells.
(TIF)

Figure S3 **Colocalization of Tim and Aurora A during G2 and M phases.** HeLa cells were synchronized by double thymidine block and release, and then assayed by indirect immunofluorescences with antibodies to Aurora (red), and Tim (green). DNA is stained with Dapi (blue) and merge images are shown in the rightmost panel. Cell cycle stages are indicated to the left of each image.
(TIF)

Figure S4 **Specificity control for Tim antibody in indirect immunofluorescence (IF) assays.** HeLa cells were transfected with siControl or siTim siRNA and then assayed by IF with anti-Tim antibody (green) or Dapi (blue). siRNA transfection efficiency was ∼80% which is reflected in the failure of some cells (∼20%) to retain green Tim signal.
(TIF)

Figure S5 **Loss of sister chromatid attachment in stable cell lines expressing Tim shRNA.** Stable HeLa derived cell lines were generated with tetracycline inducible shRNA targeting Tim or scrambled Control. ShTim or shControl cells were either untreated (−) or induced(+) with tetracycline for 48 hrs and then arrested in metaphase with colcemid for 4 hrs, followed by metaphase spread analysis.
(TIF)

Figure S6 **siTim does not evoke an intra-S phase DNA damage or mitotic spindle checkpoint response.** A) HeLa cells were transfected with siControl or siTim and then synchronized by double thymidine block and release, as shown in Figure 4. Cell extracts were isolated at various times after release from thymidine and assayed by Western blot with antibodies specific for phospho-Chk1 or total Chk1 (top two panels) or phospho-Chk2 or total Chk2 (lower two panels). Cells treated with gamma irradiation (Gy) sufficient to evoke a DNA damage response were shown in lane 1 of each panel. B) HeLa cells were treated with siControl or siTim, and further treated with or without nocodazole (60 ng/ml) for 16 hrs as indicated above each lane. Cells were then assayed by Western blot for expression of BubR1 (Abcam 8G1 ab4637) or Actin, as indicated.
(TIF)

Figure S7 **Phenotype analysis of Tim 3′UTR targeting siRNA.** A) Representative phase image of HeLa cells transfected with siControl or siTim (3′ UTR) showing change in cell morphology due to mitotic catastrophe. B) FACS cell cycle profile showing a G2/M accumulation in siTim (3′ UTR) transfected cells relative to siControl. C) Metaphase spreads of siControl and siTim 3′ UTR. D) Quantification of metaphase spreads scored for cells where >10 separated sister chromatids were observed. At least 25 metaphase spreads were scored and statistical significance was evaluated using Chi-square analysis.
(TIF)

Table S1 Summary of images from experiments represented by Fig. 3E.
(DOC)

Movie S1 Time lapsed live-cell imaging of HeLa cells expressing a GFP-H2A transfected with siControl RNA.
(MOV)

Movie S2 Time lapsed live-cell imaging of HeLa cells expressing a GFP-H2A transfected with siTim RNA.
(MOV)
