# Supplementary figures and images for: Timeless Links Replication Termination to Mitotic Kinase Activation
Source: PLoS One. 2011 May 6;6(5):e19596. doi: 10.1371/journal.pone.0019596 (PMC3089618; doi:10.1371/journal.pone.0019596)

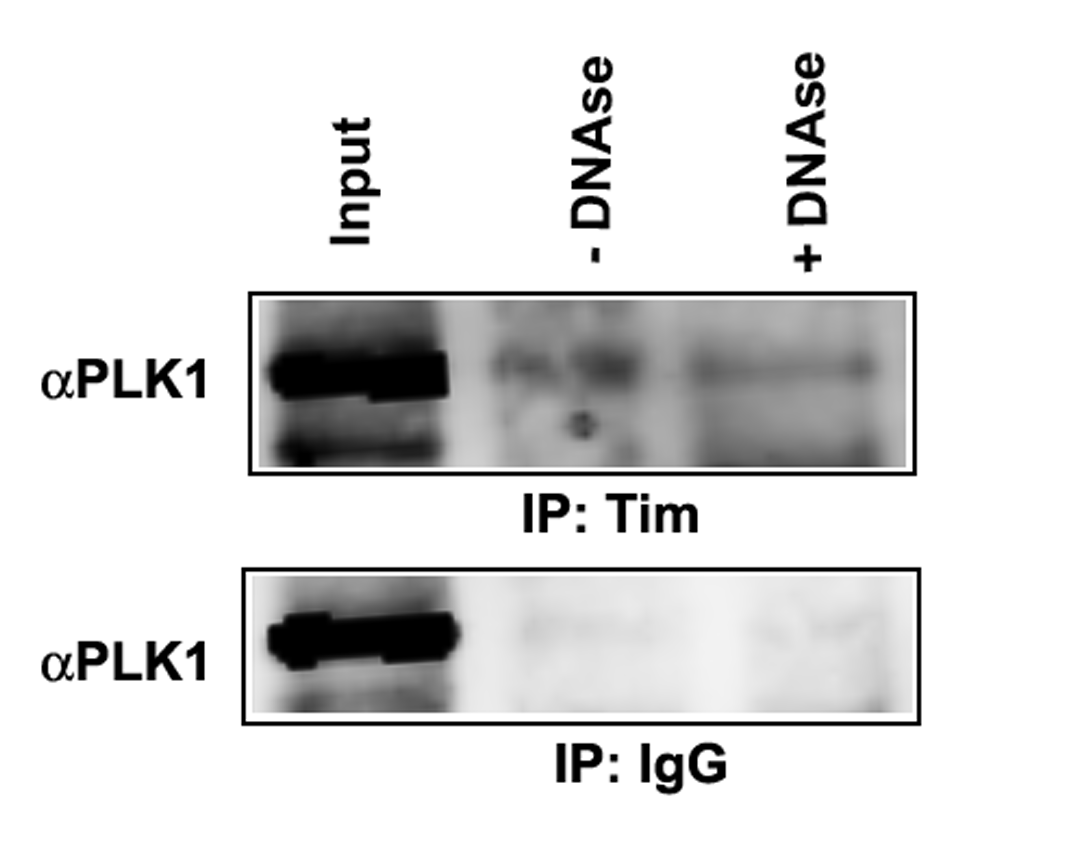

Supplement: Figure S1 — Co-immunoprecipitation of endogenous Tim with mitotic kinases. A) Asynchronous Raji cell nuclear extracts were subject to immunoprecipitation with either αTim or control IgG antibodies and then treated with 100 µg/ml DNAse I for 30 min (+) or buffer lacking DNase I (−), followed by extensive washing. Eluted proteins were then analyzed by Western blot αPlk1 (panel A) or αAurora A antibodies (panel B). (TIF) [file pone.0019596.s001.tif]

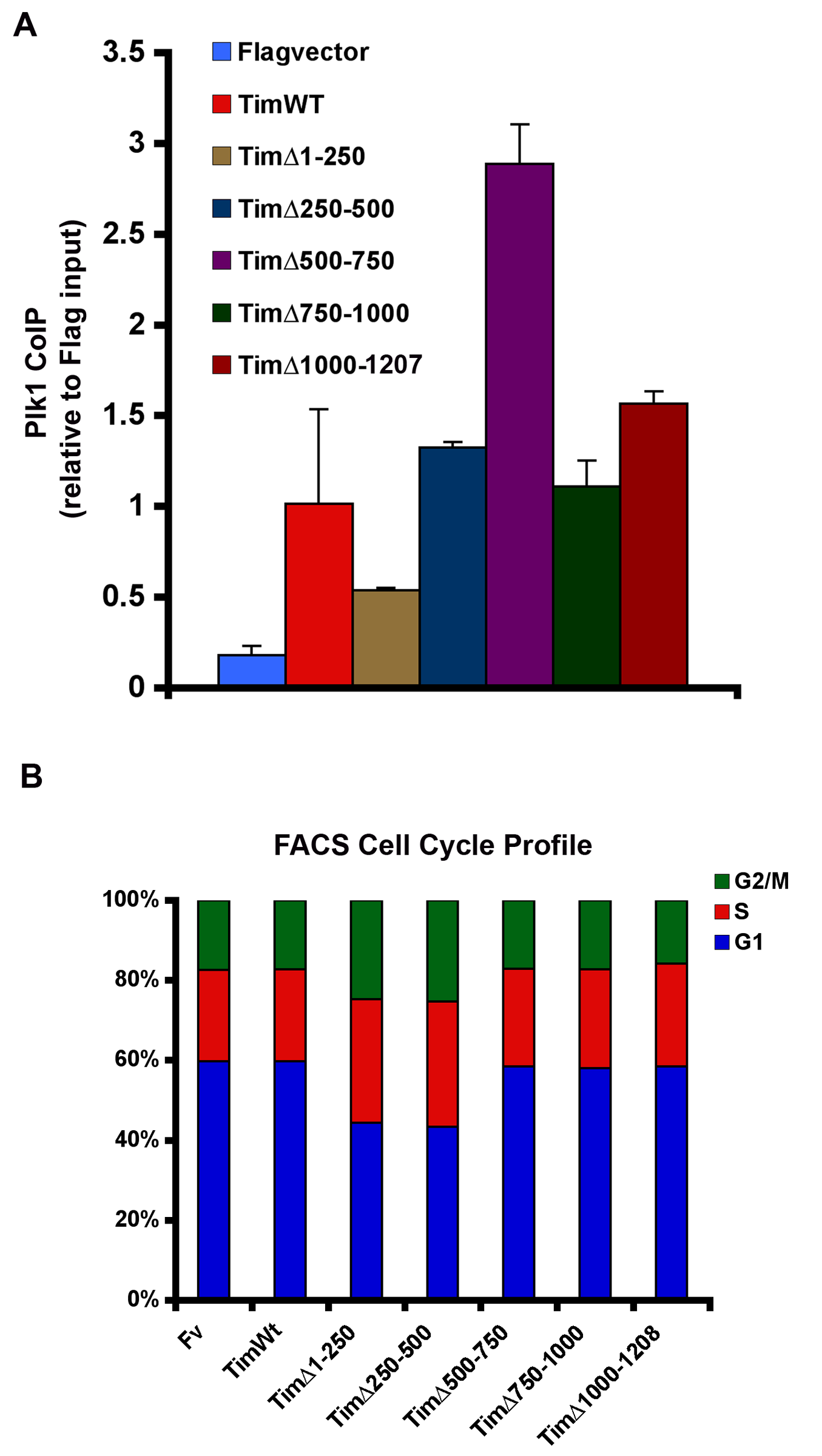

Supplement: Figure S2 — Characterization of FLAG-Tim deletion mutants. A) Quantification of at least three independnt CoIPs for Plk1 after FLAG-IP with extracts from cells transfected with FLAG vector, FLAG-Tim wt, or FLAG-Tim deletion mutants (as indicated and represented by Figure 1E). IP values were quantified as percentage of input for each Tim deletion mutant. B) Tim wt and Tim mutants were transfected and assayed for their dominant negative effects on cell cycle profile using FACS analysis of propidium iodide stained cells. (TIF) [file pone.0019596.s002.tif]

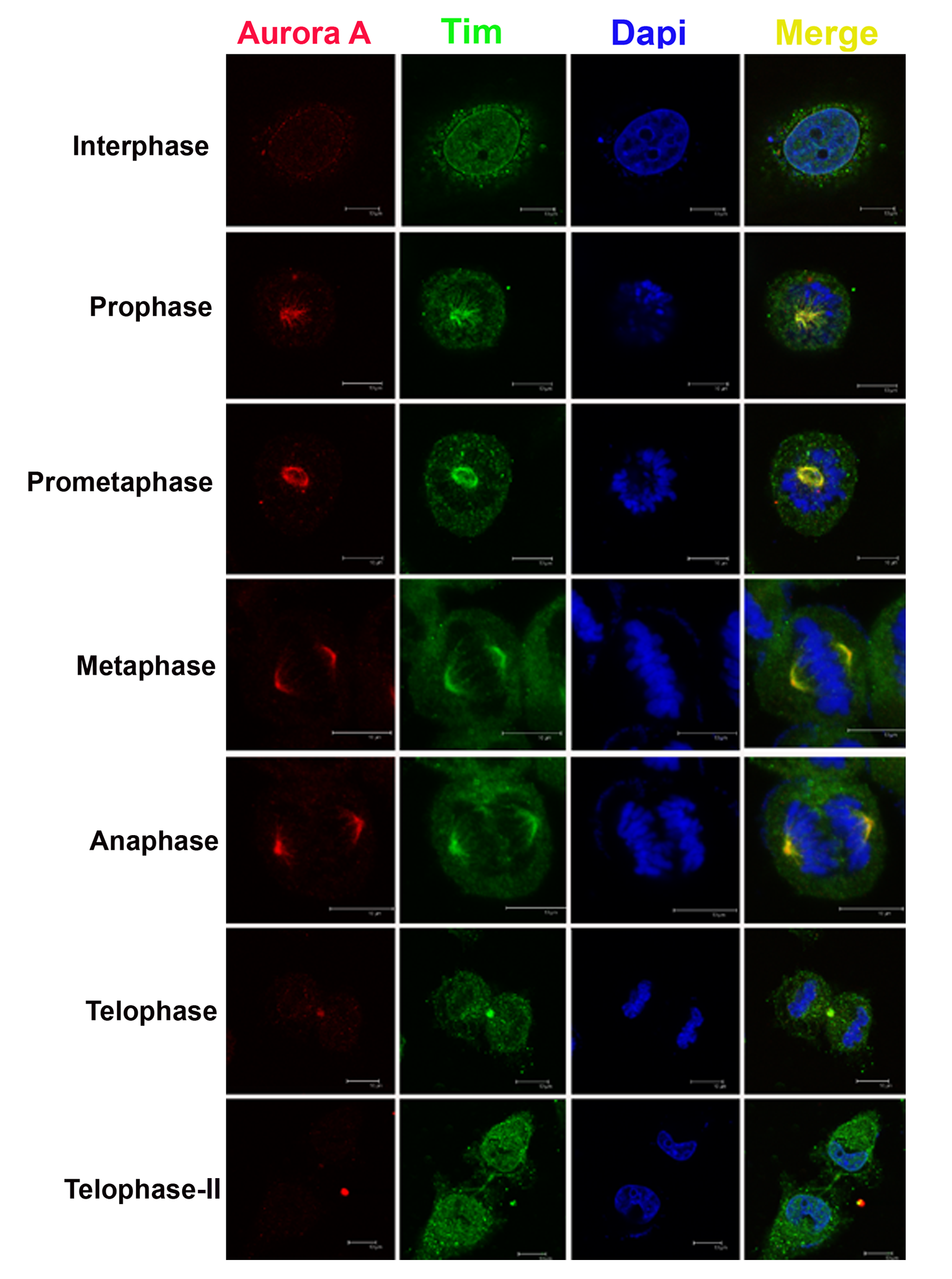

Supplement: Figure S3 — Colocalization of Tim and Aurora A during G2 and M phases. HeLa cells were synchronized by double thymidine block and release, and then assayed by indirect immunofluorescences with antibodies to Aurora (red), and Tim (green). DNA is stained with Dapi (blue) and merge images are shown in the rightmost panel. Cell cycle stages are indicated to the left of each image. (TIF) [file pone.0019596.s003.tif]

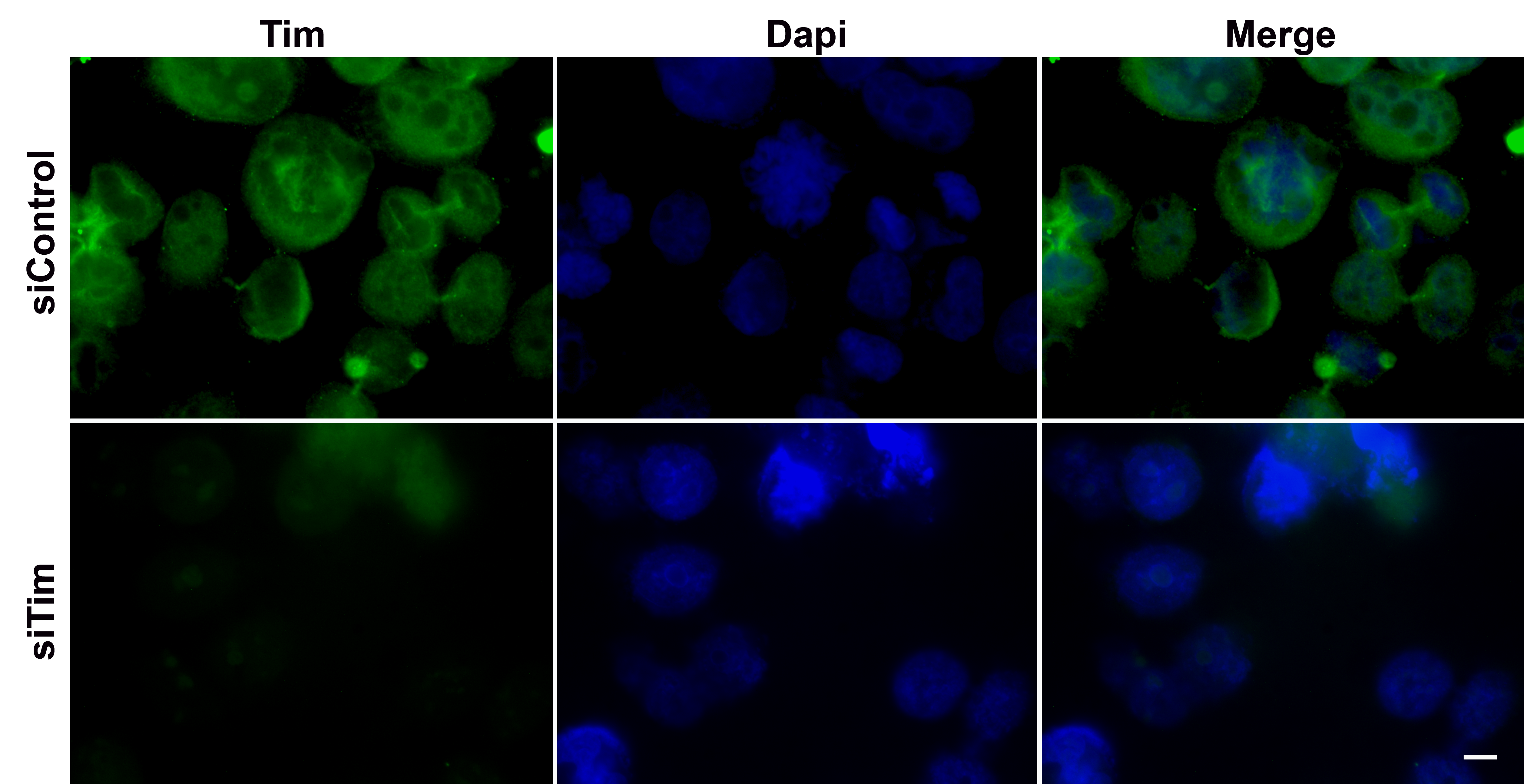

Supplement: Figure S4 — Specificity control for Tim antibody in indirect immunofluorescence (IF) assays. HeLa cells were transfected with siControl or siTim siRNA and then assayed by IF with anti-Tim antibody (green) or Dapi (blue). siRNA transfection efficiency was ∼80% which is reflected in the failure of some cells (∼20%) to retain green Tim signal. (TIF) [file pone.0019596.s004.tif]

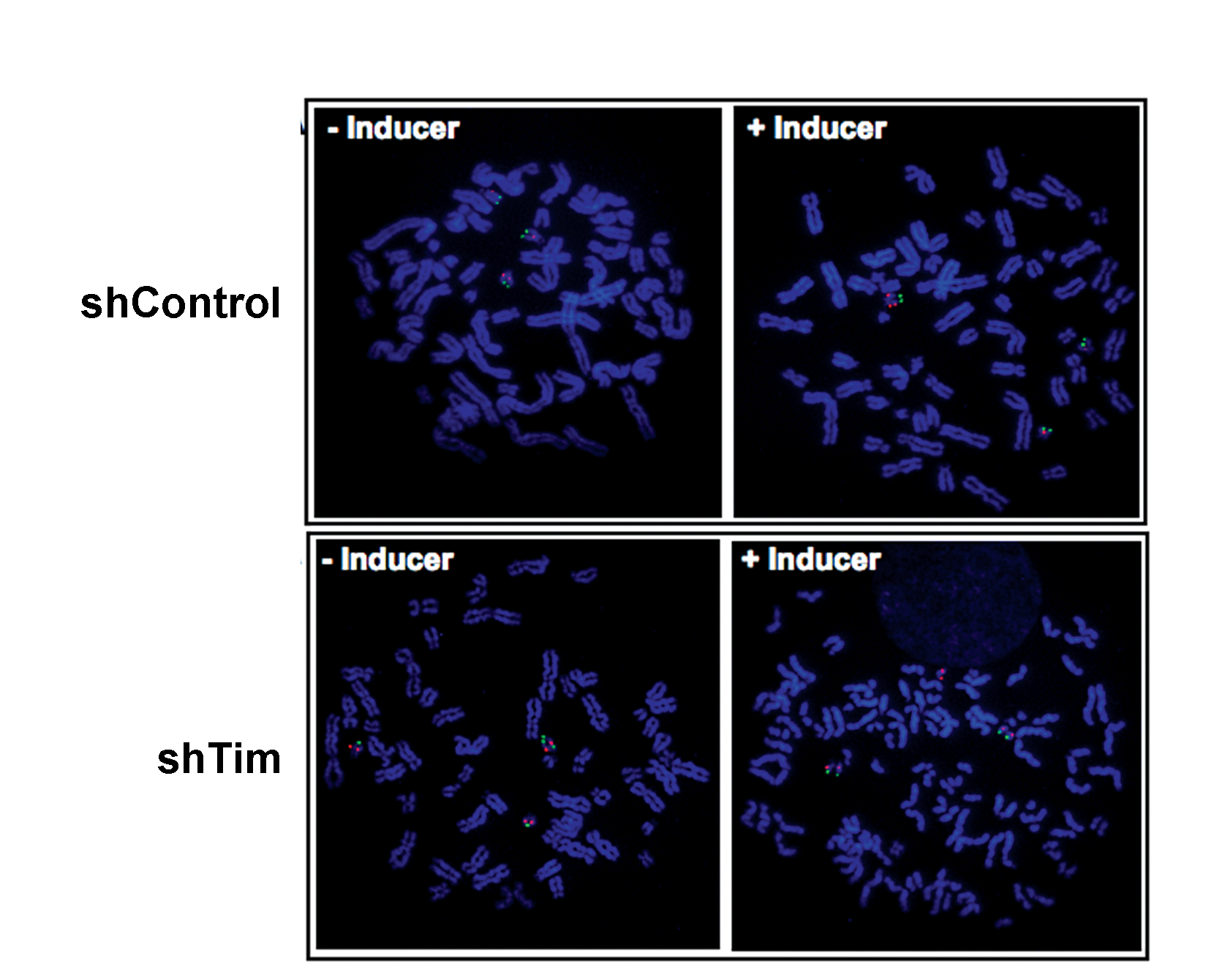

Supplement: Figure S5 — Loss of sister chromatid attachment in stable cell lines expressing Tim shRNA. Stable HeLa derived cell lines were generated with tetracycline inducible shRNA targeting Tim or scrambled Control. ShTim or shControl cells were either untreated (−) or induced(+) with tetracycline for 48 hrs and then arrested in metaphase with colcemid for 4 hrs, followed by metaphase spread analysis. (TIF) [file pone.0019596.s005.tif]

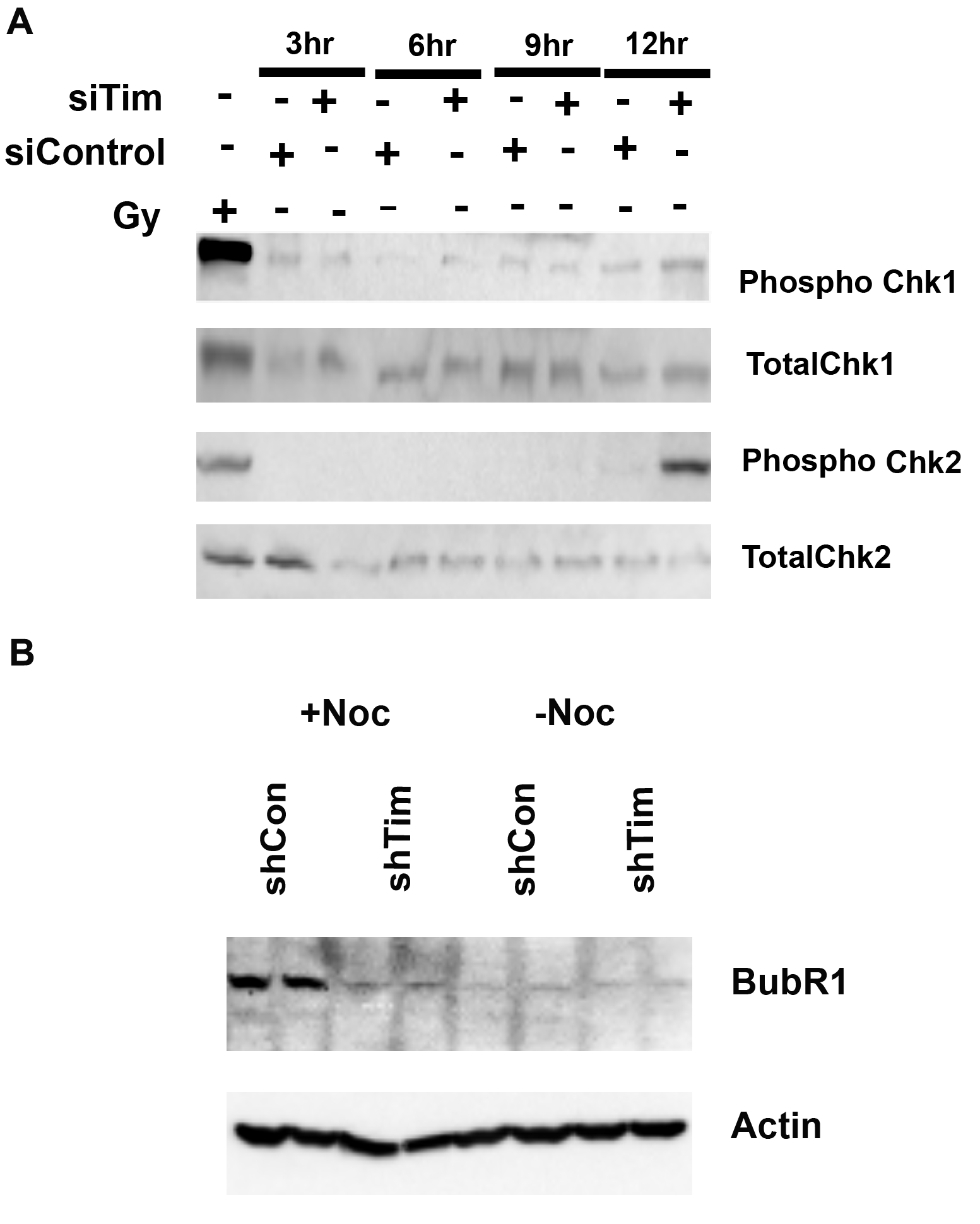

Supplement: Figure S6 — siTim does not evoke an intra-S phase DNA damage or mitotic spindle checkpoint response. A) HeLa cells were transfected with siControl or siTim and then synchronized by double thymidine block and release, as shown in Figure 4. Cell extracts were isolated at various times after release from thymidine and assayed by Western blot with antibodies specific for phospho-Chk1 or total Chk1 (top two panels) or phospho-Chk2 or total Chk2 (lower two panels). Cells treated with gamma irradiation (Gy) sufficient to evoke a DNA damage response were shown in lane 1 of each panel. B) HeLa cells were treated with siControl or siTim, and further treated with or without nocodazole (60 ng/ml) for 16 hrs as indicated above each lane. Cells were then assayed by Western blot for expression of BubR1 (Abcam 8G1 ab4637) or Actin, as indicated. (TIF) [file pone.0019596.s006.tif]

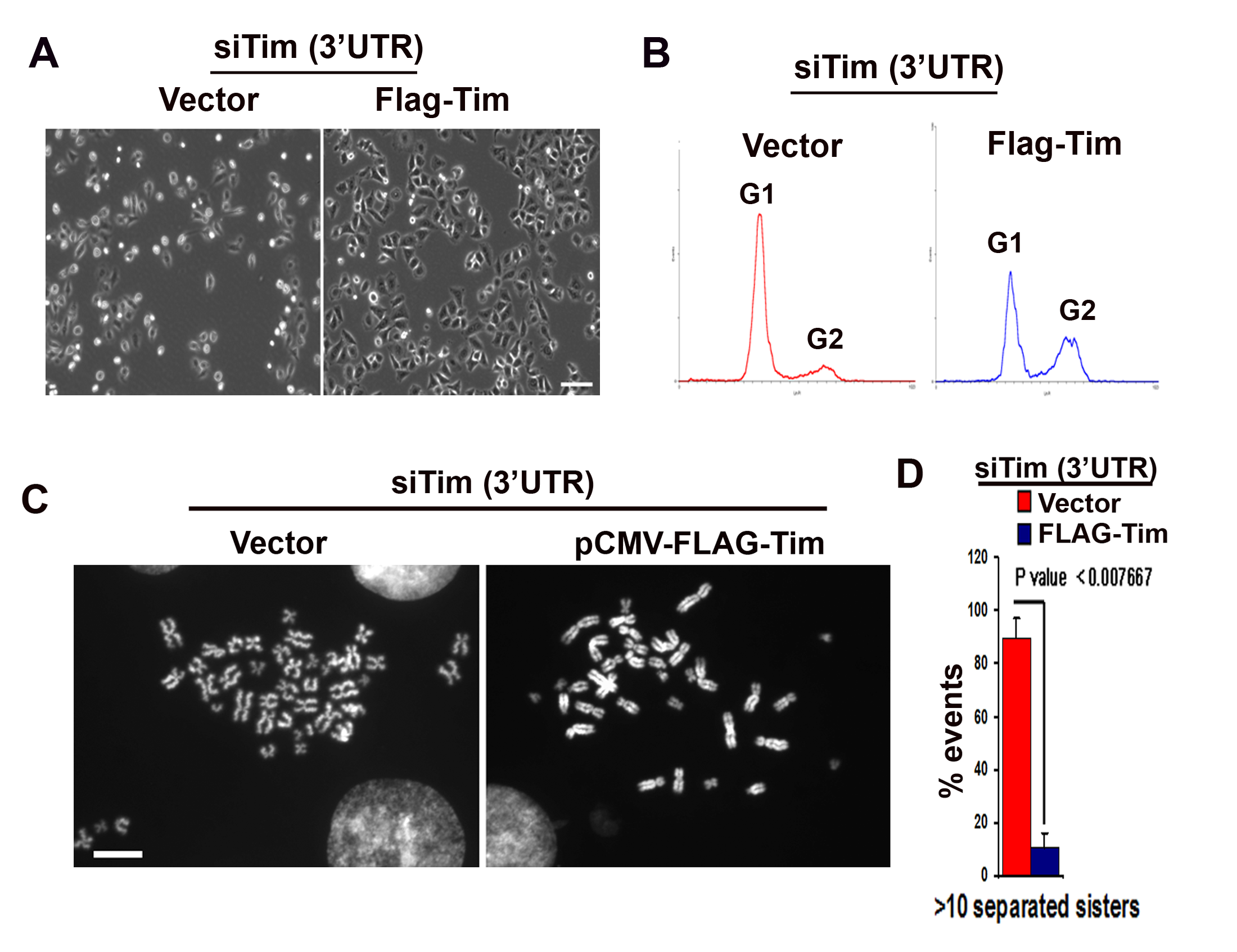

Supplement: Figure S7 — Phenotype analysis of Tim 3′UTR targeting siRNA. A) Representative phase image of HeLa cells transfected with siControl or siTim (3′ UTR) showing change in cell morphology due to mitotic catastrophe. B) FACS cell cycle profile showing a G2/M accumulation in siTim (3′ UTR) transfected cells relative to siControl. C) Metaphase spreads of siControl and siTim 3′ UTR. D) Quantification of metaphase spreads scored for cells where >10 separated sister chromatids were observed. At least 25 metaphase spreads were scored and statistical significance was evaluated using Chi-square analysis. (TIF) [file pone.0019596.s007.tif]
