# Supplementary material for: Timeless Links Replication Termination to Mitotic Kinase Activation
Source: PLoS One. 2011 May 6;6(5):e19596. doi: 10.1371/journal.pone.0019596 (PMC3089618; doi:10.1371/journal.pone.0019596)
Supplement: Table S1 — Summary of images from experiments represented by Fig. 3E. (DOC) [file pone.0019596.s008.doc]

**Supplemental Table I**

Summary of images from experiments represented by Fig. 3E.

|  | **No of metaphases counted** | **Bi-polar spindle poles** | **Defective spindle poles** |
| --- | --- | --- | --- |
| **SiCon** | **154** | **154** | **0** |
| **SiTim** | **187** | **0** | **187** |
